# Supplementary figures and images for: The piRNA cluster torimochi is an expanding transposon in cultured silkworm cells
Source: PLoS Genet. 2023 Feb 9;19(2):e1010632. doi: 10.1371/journal.pgen.1010632 (PMC9946225; doi:10.1371/journal.pgen.1010632)

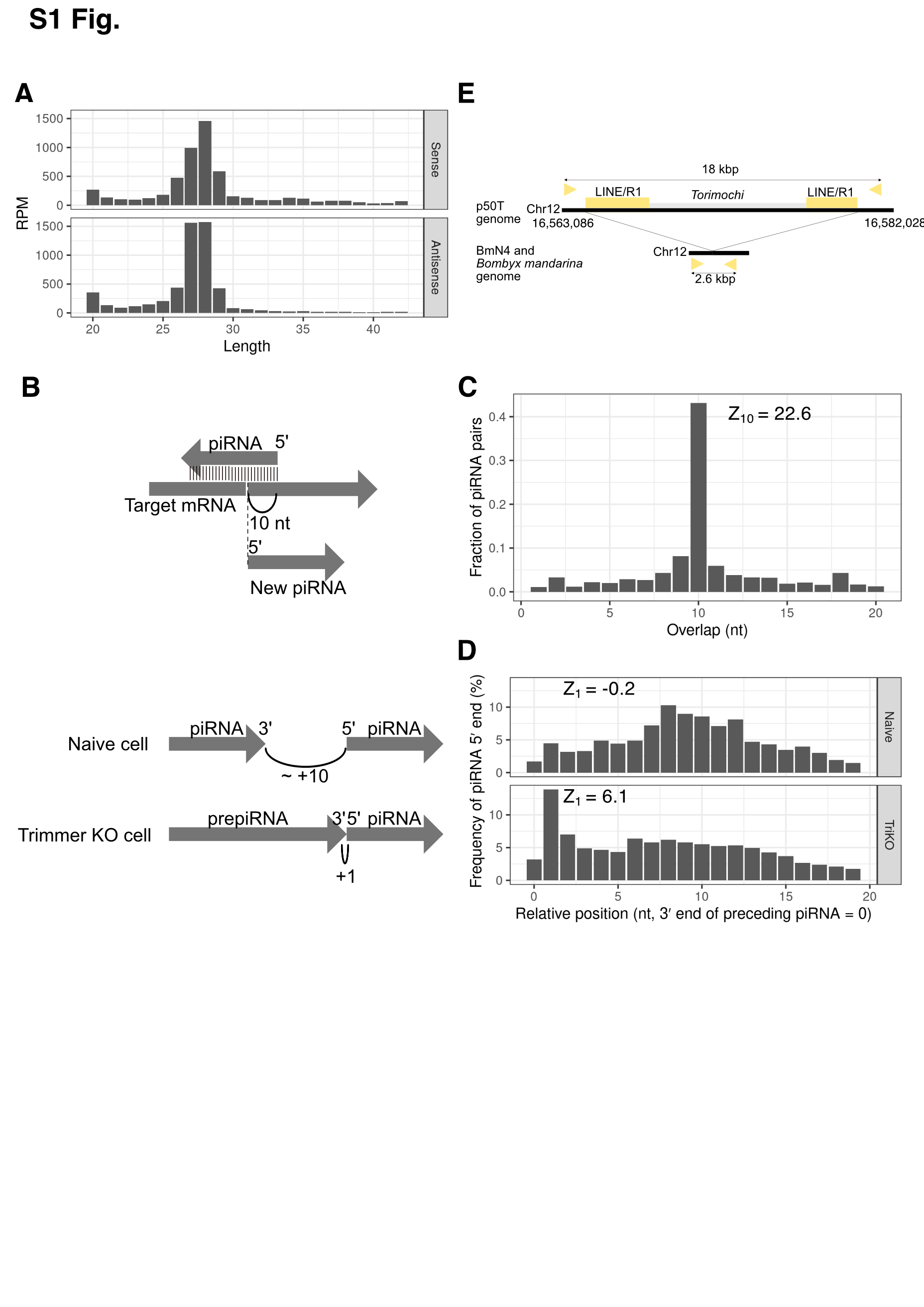

Supplement: S1 Fig — (A) The length distribution of torimochi-derived small RNAs in BmN4 cells. (B) Schematics for the ping-pong signature (C) and the phasing signature (D). (C) The fraction of 5’-5’ overlapped piRNAs (y-axis) at the indicated length (x-axis) for torimochi-derived piRNAs in the small RNA libraries from BmN4 cells. The Z-scores at the 10 nt overlap (ping-pong signature) are shown in the top-right of the graphs. (D) The fraction of 3’-5’ distance for torimochi-derived piRNAs in naive BmN4 cells or BmN4 cells knocked out for Trimmer, the exonuclease that trims the 3’-end of precursor piRNAs for maturation (TriKO). Z1 denotes the z score at position 1. A strong tail-to-head phasing signature (at position = 1) was detected in torimochi-derived piRNAs from TriKO cells. (E) In the chr12 region of the p50T genome, torimochi is nested within another transposon, LINE/R1. This LINE/R1 element is extremely GC-rich and repetitive, which likely hinders the PCR amplification of the full-length sequence in Fig 1F. On the other hand, a strong band was detected at ~2.0 kb for BmN4 (Fig 1F), which indicates the absence of both torimochi and LINE/R1 in this region. (TIFF) [file pgen.1010632.s001.tiff]

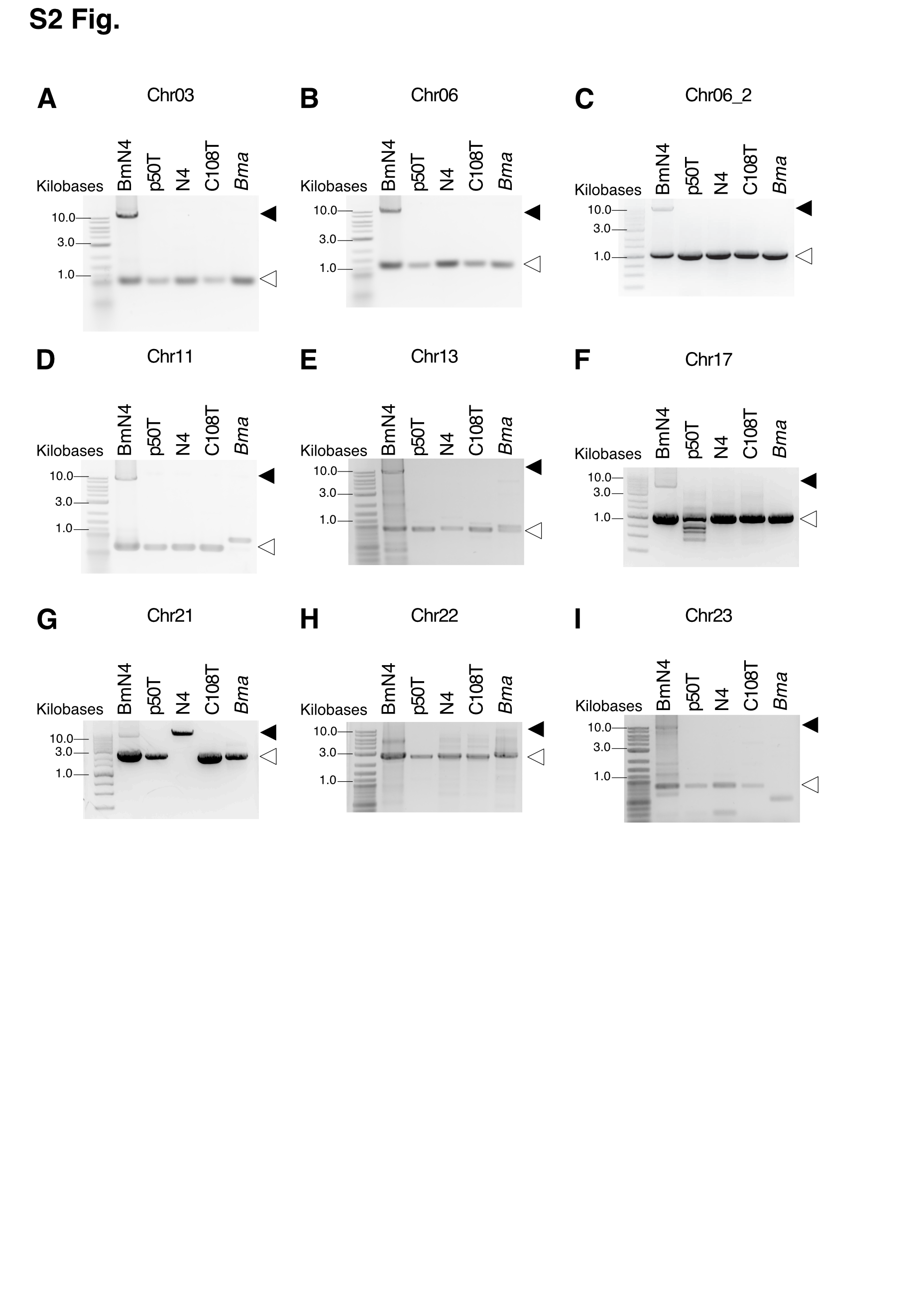

Supplement: S2 Fig — (A–I) Genomic PCR of the torimochi inserts newly identified in BmN4 cells. The genome DNAs of BmN4 cells and silkworm strains p50T, N4, and C108T and B. mandarina (Bma) were used. Black and white arrows indicate the band lengths with and without torimochi, respectively. (G) The genome of the N4 strain has a torimochi insertion at the same site. (TIFF) [file pgen.1010632.s002.tiff]

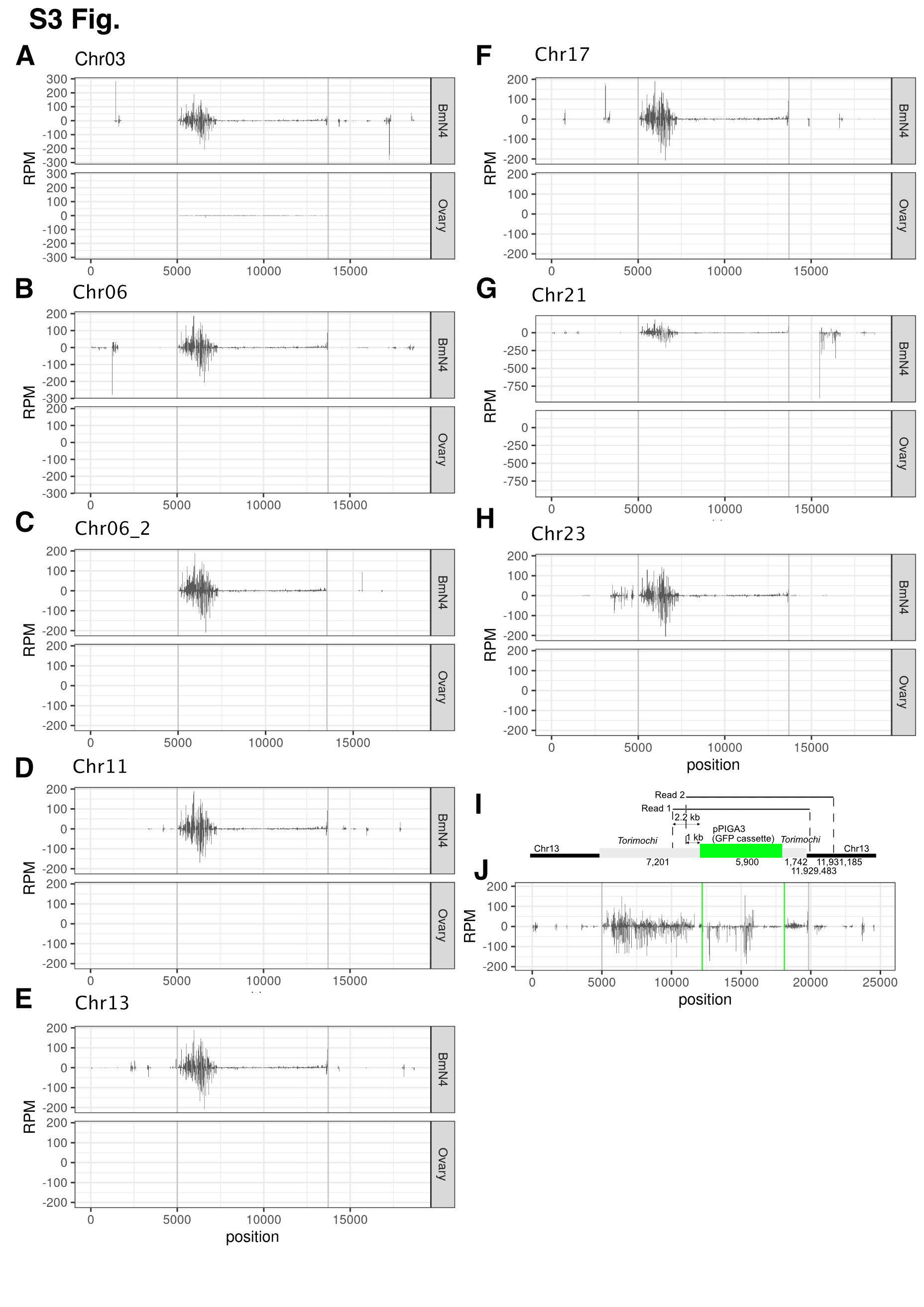

Supplement: S3 Fig — (A–H) Distribution of torimochi-derived piRNAs in BmN4 cells and ovaries. The boundaries of torimochi are shown by gray lines. (I) Schematic diagram of torimochi, GFP transgene, and the outside genomic regions on chromosome 13. The MinION reads that span these regions are shown by black lines. (J) piRNA production from the torimochi copy on chromosome 13 in BmN4 cells and ovaries (TIFF) [file pgen.1010632.s003.tiff]

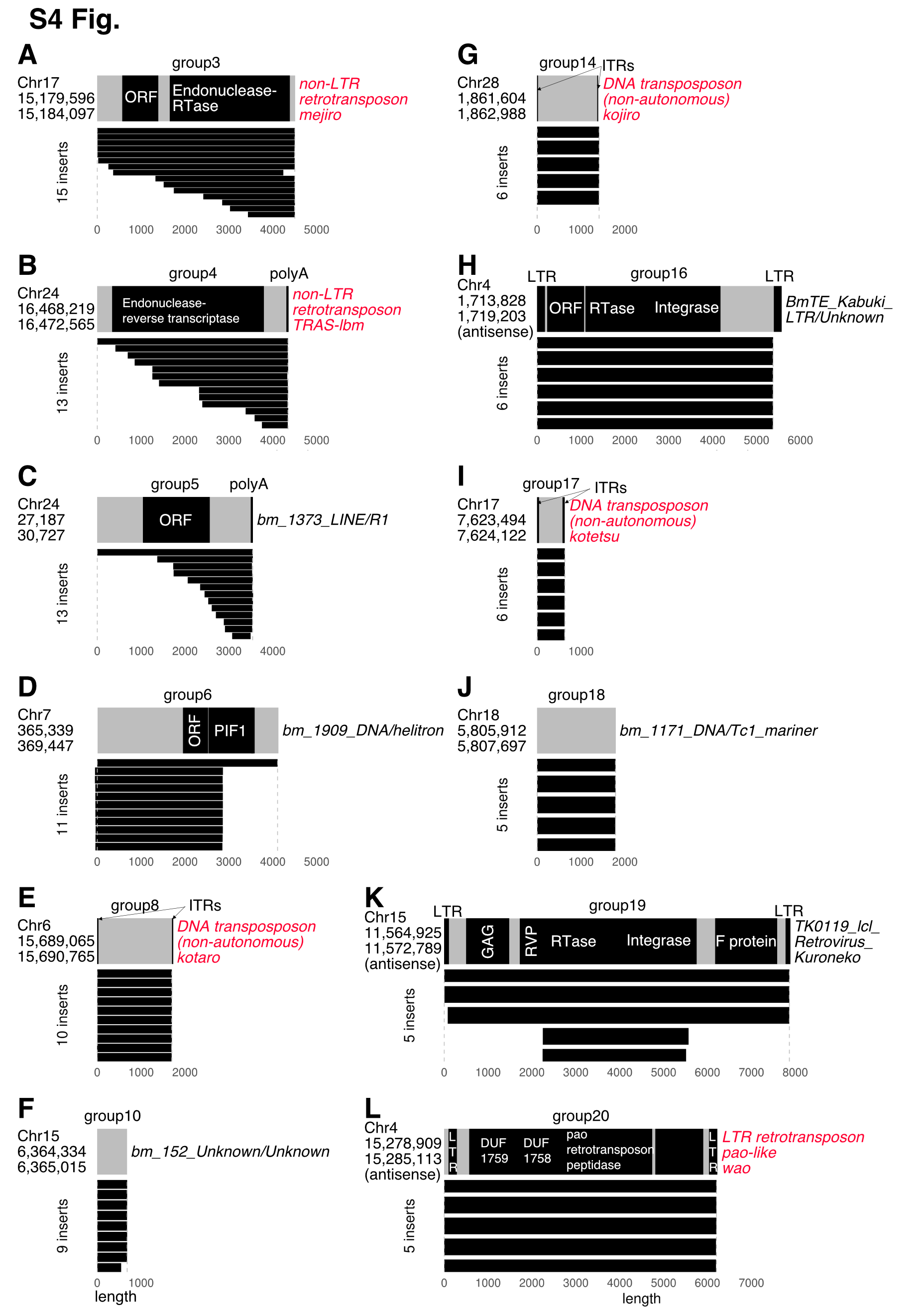

Supplement: S4 Fig — (A–L) Domain structure of the representative sequence of each group (top), the region where it is originally annotated in the p50T genome (left), and the lengths of the inserts found in BmN4 cells (bottom). ORF: ORFs that could not be annotated by Pfam. Names of newly identified transposons are indicated in red. (TIFF) [file pgen.1010632.s004.tiff]

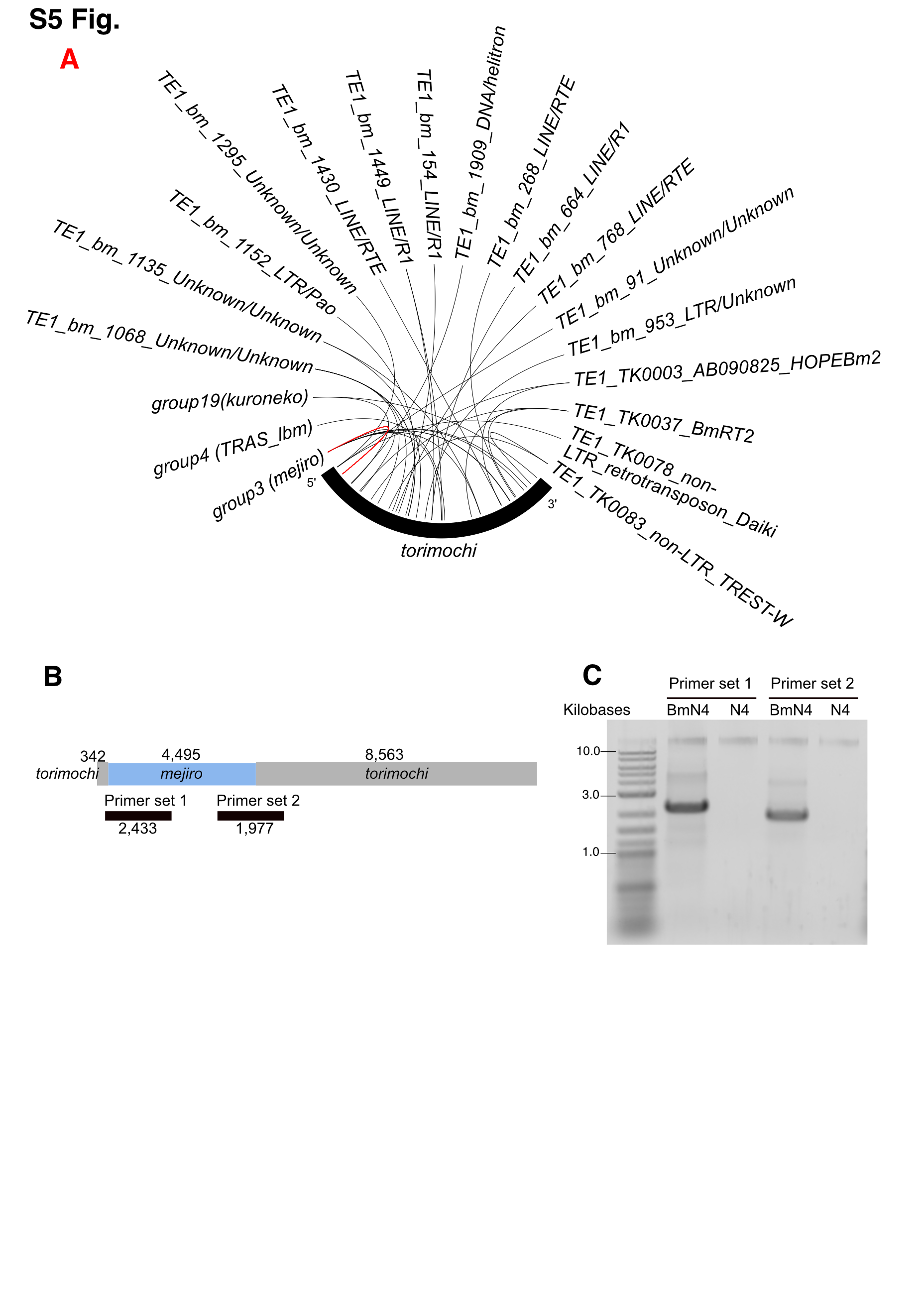

Supplement: S5 Fig — (A) Transposons inserted in torimochi and their locations. Transposons with at least one end (within 100 bp) forming a junction with a sequence inside torimochi were considered to be inserted into torimochi. Seven out of the 36 inserts accounted for mejiro (S4A Fig). The red line shows the insertion whose presence was confirmed by genomic PCR in S5B and S5C Fig. (B) The insertion of mejiro within torimochi shown as red in S5A Fig. The primer sets used in C are shown by black bars. (C) Genomic PCR shows the transposon insertion specifically in BmN4 cells. (TIFF) [file pgen.1010632.s005.tiff]

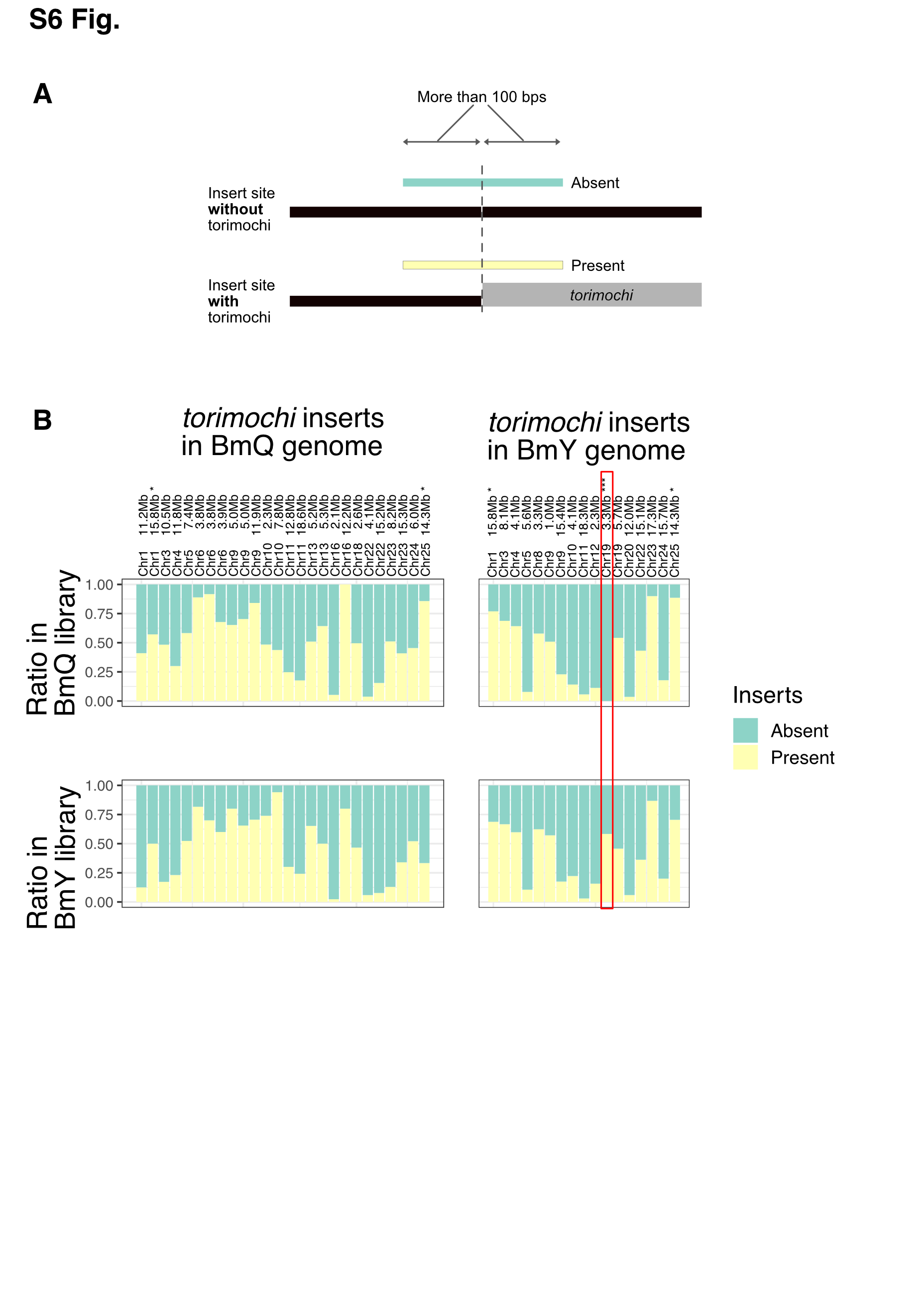

Supplement: S6 Fig — (A) Definition of torimochi insertion. The presence or absence of the reads that cover at least 100 bps at both sides of a given junction site was used as a criterion in (B). (B) The torimochi insertion sites identified in the BmQ (left) or BmY (right) genome. The ratio of MinION reads from BmQ (top) or BmY (bottom) cells with or without torimochi sequence was plotted for each identified insertion site. (TIFF) [file pgen.1010632.s006.tiff]
