# Supplementary material for: The piRNA cluster torimochi is an expanding transposon in cultured silkworm cells
Source: PLoS Genet. 2023 Feb 9;19(2):e1010632. doi: 10.1371/journal.pgen.1010632 (PMC9946225; doi:10.1371/journal.pgen.1010632)
Supplement: S1 Table — (PDF) [file pgen.1010632.s007.pdf]

(1) Genome extraction and genomic PCR		
chr11 p50T F	GACTTAAATTTACAAACGATGCTAGTATGAAT	
chr11 p50T R	AAAAGAGAAGCCTATCTACAGACATGTTAGAC	
chr12 p50T F	AGAGGTGCCTAACAATCTGACAAGCTT	
chr12 p50T R	GCATAAGGTGAGGTGTCTTTGAC	
chr24 p50T F	TCTTTAGGACTATGTAGGTTGTTGTTAGTTAT	
chr24 p50T R	ATAGTTGCTACTTTAAGCTCATCTCATCAT	
chr03 BmN-4 F	CGTAGAAACTCGTAGCCGTCTTA	
chr03 BmN-4 R	ATGCAAGTGTAAAAGAGCGTAGG	
chr06 BmN-4 F	TGTCCTCGCGAGACTATTAAAAC	
chr06 BmN-4 R	AATCCACCGAAATCTTTGTACCT	
chr06_2 BmN-4 F	TTTACCAAGGACTGATGCTGATT	
chr06_2 BmN-4 R	TTACCGGAAACTAAGCACATGAT	
chr11 BmN-4 F	GATGGCTTTACGTTTATGCTCTC	
chr11 BmN-4 R	CCACATCCAGGTTCCAAATAATA	
chr13 BmN-4 F	AGTACGACATGGAACTGATCACC	
chr13 BmN-4 R	CATGTCTCAAGGTGGGTTTTTAC	
chr17 BmN-4 F	AATGCTCGTGCTAACAAATTCAT	
chr17 BmN-4 R	CAACTGTTTGTGCAAGTCTGAAG	
chr21 BmN-4 F	TTATGCACCTCTTTCCAACAGAT	
chr21 BmN-4 R	TTTTTATAATCGTCAATAAAGGCTAAATCTATATC	
chr22 BmN-4 F	GTTAAGTGCTCCCGTATGACTTG	
chr22 BmN-4 R	TATATCTTAAATGTAGCGTCTATTTTTGTAGTCAA	
chr23 BmN-4 F	GTTAACCGTGAAACCACAGACAT	
chr23 BmN-4 R	AGAAAATCGTCCTTTTTGTAGGC	
torimochi_300F	AAAAGCTCATCGCCGTATCCTGAAG	Figure S5
mejiro_2099R	TTTAAGAAACACCCAAACCGAAAATG	Figure S5
mejiro_3026F	TGAGTGGGAGTTACAGAGTGTTGGAG	Figure S5
torimochi_795R	GCGACGAATATTGTAGCTCGAAGTCT	Figure S5

(2) Quantitative PCR for measuring the copy number of *torimochi*	
torimochi F	AAGTCTTCTCCTATCCTCCTAAATCAGT
torimochi R	TTCAGTGGATATAGTATGTGGATACAGTG
rp49 F	CATTCAGGAATTGAACAGTGACA
rp49 R	TGAGGGACAAGACACACCTTAAT

(3) Cloning of *torimochi* and phylogenetic tree analysis
*Indicated numbers are the positions of each primer in torimochi on chromosome 3. **Primers listed in (1) are used as "outside" primers for PCR.

sequence F1	CAATATGCGCTTAAAATCGAAAC	1436..1458	also used as an "inside" primer for PCR (chr03, 06, 06_2, 11, 13, 21, 23)
sequence F2	CAAAAACCAGTCCATTCCTG	1885..1904	
sequence F3	ACCCACTGAATCCATTCTGCA	2415..2435	
sequence F4	TGACGATCTCTTAGTAGCTAACTG	2928..2951	
sequence F5	TCCCCTTGGTCCTCACCTAT	3406..3425	
sequence F6	CTTCAATCTCAAACTACAACCT	3852..3873	
sequence F7	GGCCATACCTCTATGGCAGA	4376..4395	also used as an "inside" primer for PCR (chr17)
sequence F8	GAGGACCTTACCGTGAATCT	4669..4688	
sequence F9	TTATAAGTAAGGACAAAACCCGATATAGTAAT	7482..7513	also used as an "inside" primer for PCR (chr03, 06, 06_2, 11, 13, 23)
sequence F10	CGATAACAATAAATGGATATTTGTTCAA	7699..7726	
sequence F11	CCCGATAACATGTAAAGGTT	7810..7829	
sequence F12	CTTTATGTAAACTGTATCGCTATTATAAAA	8139..8168	
sequence F13	ACCCCTATAATAACGGCATC	8327..8346	
sequence F14	TATCCTCCTAAATCAGTGCGCG	8626..8647	
sequence R1	CGATTAAGAACAAGGGTTGATTG	889..911	
sequence R2	TCGCGAGACATGTTAGGATTATT	1724..1746	also used as an "inside" primer for PCR (chr03, 06, 06_2, 11, 13, 17, 21, 23)
sequence R3	GGGTTTCCATTATTTCAGGAAC	5077..5098	also used as an "inside" primer for PCR (chr17)
sequence R4	CCAGCTATAGGAATATCGTGG	5457..5477	
sequence R5	TACTCTTTGAGTGTGGAATGGC	5963..5984	
sequence R6	GAACTAATTGCTAGGATTGGCAC	6455..6477	
sequence R7	TGGATCAATTTATGTTTTAGTAACGACGC	6686..6714	
sequence R8	TTGGTGTAATTGAGACGGCG	7203..7222	
sequence R9	CATAATTGATTGATTTACATTGGCTTAGGG	7521..7550	
sequence R10	GTGGTTCCCACACATCTATGATT	7675..7697	also used as an "inside" primer for PCR (chr03, 06, 06_2, 11, 13, 21)

(4) Cloning of newly identified transposons		
mejiro_out_L	TATTAAGTTTTGGCTTTACCTTC	also used as an "outside" primer for PCR
mejiro_out_R	AACTTCACTCGCACGTCTTCTAC	also used as an "outside" primer for PCR
mejiro_in_F	TAACGTTCCGCTCGAAATAAATA	also used as an "inside" primer for PCR
mejiro_in_R	CGTCTCTATCCTGTGCTACGTCT	also used as an "inside" primer for PCR
mejiro_seq_R1	TGGGCCAATGGATTCGAGTTTGA	
mejiro_seq_R2	ATTTGTTGACTATTTTGTATAAGG	
mejiro_seq_R3	ATAAAGCCCACCCCATATTGTCC	

mejiro_seq_R4	AGGTTAGTTTGACTGAGAAATTT	
TRAS-lbm_outR	ACGTGGACTTGGGAACAAGGACA	also used as an "outside" primer for PCR
TRAS_lbm_outL	TTAACTATACTAGAGGTATACTTAA	also used as an "outside" primer for PCR
TRAS-lbm_in_F	GAAGTGTCAAGAGAAAAGCCAAA	also used as an "inside" primer for PCR
TRAS-lbm_in_R	GCTACGAGGAATACGAGGAATTT	also used as an "inside" primer for PCR
TRAS-lbm_seq_F1	ATCGGTTGGAGATACTGAACTGC	
TRAS-lbm_seq_F2	AATAGTACAAAAAGTAAACTTTAGAAA	
TRAS-lbm_seq_R1	ACACCGTGTTCACATGTTTCACC	
TRAS-lbm_seq_R2	AGAGCCAGGAGTTCAGCTTGGTA	
TRAS-lbm_seq_R3	CTCTCCACTAAACACGAGCACTA	
kotaro_out_L	AAATCCTGTCATGAGTCCTGGT	also used as an "outside" primer for PCR
kotaro_out_R	GATGTCAGTTTGGTGGAAATAGG	also used as an "outside" primer for PCR
kotaro_in_F	GAAATCAGCAACAATCCAGCTAC	also used as an "inside" primer for PCR
kotaro_in_R	AACGCGTTGAAATAGAATACAGG	also used as an "inside" primer for PCR
kotaro_seq_R1	GTGATTTATGTATTAGTAATGAC	
kojiro_out_L	GATCTAGTTTCACGCGAGTGTCT	also used as an "outside" primer for PCR
kojiro_out_R	TTAGCGCCACCACAATGTAA	also used as an "outside" primer for PCR
kojiro_in_F	CGAAGCGACAATTGTTCAGA	also used as an "inside" primer for PCR
kojiro_in_R	CCCCTAGATCCCCTAAACGA	also used as an "inside" primer for PCR
kojiro_seq_F1	TTTCAACTTCCCTATTATTAGAG	
kotetsu_out_L	ACAGTTAATTGGACTGCATCGTT	also used as an "outside" primer for PCR
kotetsu_out_R	GCACAATTCGATCTCAAAGTAGG	also used as an "outside" primer for PCR
kotetsu_in_R	CTCGGATCTAACGTGCTAGTGTT	also used as an "inside" primer for PCR
kotetsu_in_F	AGTGTCGAAAATGGATAACGAAA	also used as an "inside" primer for PCR
kotetsu_seq_F1	ATTGCTGAAGTCCATTGGACGGT	
kotetsu_seq_F2	CGCACTGATGCCGTCTGTAAATA	
wao_out_L	ACACTACAACTTCTGCCCCATAA	also used as an "outside" primer for PCR
wao_out_R	GTAGGTATCATCACGAGCGAATC	also used as an "outside" primer for PCR
wao_in_F	GCAAGTGATCAGTGCCTTAGAGT	also used as an "inside" primer for PCR
wao_in_R	TGACAAAGGGCGAGAATTTAATA	also used as an "inside" primer for PCR
wao_seq_F1	GGATTCGACGAGAACGATGACTG	
wao_seq_F1.5	ACGAGGAACGAACCTGCTCCTGC	
wao_seq_F2	AGATACAAGTGGCTGAAATACAA	
wao_seq_R1	CCTGATCTTCCACTTGGTTCGTA	
wao_seq_R2	GTTGTATTGTTTACAATTTGTTC	
wao_seq_R3	TATTTCCTTTAAAAATCTTAATG	
wao_seq_R4	ACTACTTTCTTTAATAACACCGT	
wao_seq_R5	CCACTATCCAGAAGCGCACGAGC	
