# Supplementary material for: The piRNA cluster torimochi is an expanding transposon in cultured silkworm cells
Source: PLoS Genet. 2023 Feb 9;19(2):e1010632. doi: 10.1371/journal.pgen.1010632 (PMC9946225; doi:10.1371/journal.pgen.1010632)
Supplement: S2 Table — The longest sequence in each group as representative sequence was annotated by BLASTn. Green or gray rows are those that could not be illustrated in S3 Fig due to many variations in the sequence. Green group’s representative sequences are LINE/SINE and gray are others. (PDF) [file pgen.1010632.s008.pdf]

	insert ID	source chromosome	source_start	source_end	length	BLAST target	BLAST length	BLAST score	newly identified
group_001	459	Bomo_Chr6	8711020	8717375	6356	TE1_bm_275_SINE/Bm1			
group_002	390	Bomo_Chr12	16568093	16577026	8934	121TEs_TK0037_BmRT2	2406	0	
group_003	456	Bomo_Chr17	15179596	15184097	4502	BmTE_bm_2220_LINE/RTE	262	e-147	novel
group_004	83	Bomo_Chr24	16468219	16472565	4347	BmTE_bm_1099_LTR/Unknown	68	4.00E-96	novel
group_005	302	Bomo_Chr24	27187	30727	3541	BmTE_bm_1373_LINE/R1	1600	0	
group_006	701	Bomo_Chr7	365339	369447	4109	BmTE_bm_1909_DNA/helitron	all	0	
group_007	216	Bomo_Chr21	6329385	6331469	2085	TE1_bm_1012_LINE/Jockey			
group_008	554	Bomo_Chr6	15689065	15690763	1699	BmTE_bm_549_LTR/Unknown	15	0.23	novel
group_009	399	Bomo_Chr16	5949754	5956702	6949	TE1_bm_8_LINE/R1			
group_010	144	Bomo_Chr15	6364335	6365015	681	BmTE_bm_152_Unknown/Unknown	all	0	
group_011	177	Bomo_Chr24	1527202	1528728	1527	TE1_bm_77_SINE/Unknown			
group_012	308	Bomo_Chr10	9162068	9164139	2072	BmTE_bm_422_DNA/helitron	all	0	
group_013	373	Bomo_Chr2	122197	123606	1410	BmTE_bm_1518_Unknown/Unknown	all	0	
group_014	78	Bomo_Chr28	1861604	1862988	1385	BmTE_bm_2154_Unknown/Unknown	235	e-131	novel
group_015	642	Bomo_Chr14	6582404	6583281	878	BmTE_bm_98_SINE/Bm1			
group_016	81	Bomo_Chr4	1713828	1719203	5376	BmTE_Kabuki_LTR/Unknown	all	0	
group_017	249	Bomo_Chr17	7623492	7624122	631	121TEs_TK0030_BmRT1	16	0.11	novel
group_018	458	Bomo_Chr5	5805912	5807697	1786	BmTE_bm_1171_DNA/Tc1_mariner	1200	0	
group_019	88	Bomo_Chr15	11564925	11572789	7865	121TEs_TK0119_lcl_Retrovirus_Kuroneko	all	0	
group_020	304	Bomo_Chr4	15278909	15285113	6205	BmTE_bm_2017_LTR/Pao	169	2.00E-91	novel
