# Supplementary material for: The piRNA cluster torimochi is an expanding transposon in cultured silkworm cells
Source: PLoS Genet. 2023 Feb 9;19(2):e1010632. doi: 10.1371/journal.pgen.1010632 (PMC9946225; doi:10.1371/journal.pgen.1010632)
Supplement: S3 Table — The "old_area" represents the length of homologous regions in the p50T genome, and the "new_area" represents the total length of the inserts identified in this study. (PDF) [file pgen.1010632.s009.pdf]

	old_area	old_sites	new_area	new_sites	new_average_length
group_001	30903672	72102	121712	80	1521.4
group_002	30254	10	217890	26	8380.384615
group_003	18667	28	50269	15	3351.266667
group_004	19903	21	32846	13	2526.615385
group_005	64078	53	17564	13	1351.076923
group_006	206997	285	40010	11	3637.272727
group_007	14513775	8961	29703	11	2700.272727
group_008	7928	8	16967	10	1696.7
group_009	8251159	10416	31525	10	3152.5
group_010	221915	360	5804	9	644.8888889
group_011	6502456	11215	5825	8	728.125
group_012	761424	837	10083	8	1260.375
group_013	810726	2150	5360	7	765.7142857
group_014	10223	10	8350	6	1391.666667
group_015	180769	303	4449	6	741.5
group_016	31221	17	32245	6	5374.166667
group_017	2096	4	3751	6	625.1666667
group_018	17140	17	8911	5	1782.2
group_019	18828	12	30126	5	6025.2
group_020	19682	7	30994	5	6198.8
