# Supplementary material for: The piRNA cluster torimochi is an expanding transposon in cultured silkworm cells
Source: PLoS Genet. 2023 Feb 9;19(2):e1010632. doi: 10.1371/journal.pgen.1010632 (PMC9946225; doi:10.1371/journal.pgen.1010632)
Supplement: Attachment — Submitted filename: 128007_1_rebuttal_2456453_rhty9v-1.pdf [file pgen.1010632.s010.pdf]

Revision Plan

Review COMMONS

Manuscript number: RC- 2022-01542
Corresponding author(s): Keisuke Shoji and Yukihide Tomari

1. General Statements [optional]

We thank both the Reviewers for critical comments and constructive suggestions, which help us to significantly improve our manuscript. As detailed below, we are confident that we will be able to address all the points raised by the Reviewers and the revised manuscript will be suitable for publication.

2. Description of the planned revisions

Reviewer #1 (Evidence, reproducibility and clarity (Required)):

Summary

PIWI-interacting RNAs (piRNAs) are required for transposon repression and are transcribed from discrete genomic loci termed piRNA clusters. Torimochi was identified as a piRNA cluster in silkworm in 2012, but the incomplete genome assembly hindered its further characterisation. Here, Shoji and colleagues characterised torimochi using the current, recently improved, genome assembly, combined with long-read (MinION) and Sanger sequencing. This reveals that torimochi is a regular Gypsy LTR transposon. Comparison of copy number across strains reveals that torimochi has been particularly active in the BmN4 cell line, showing different insertions between strains. Moreover, piRNAs are produced from multiple torimochi copies across the genome. Lastly, the authors show that torimochi has an open chromatin conformation. The authors propose that torimochi may be a young and still growing piRNA cluster, capable of both trapping other transposable elements and transgenes and of producing piRNAs.

Major comments

How are the torimochi-derived piRNAs produced? Which part of the piRNA pathway are required for their production? Determining this would significantly strengthen the study and potentially support the idea that torimochi is a "young and still growing piRNA cluster". Currently, it is unclear what evidence there is for torimochi acting as a piRNA cluster rather than a regular LTR transposon.

We thank the Reviewer for raising this important point. We have now re-analyzed our piRNA sequencing data and confirmed that 1) production of *torimochi*-derived piRNAs requires Siwi, the core PIWI protein component in silkworms and 2) *torimochi*-derived

piRNAs show the ping-pong signature, as observed for other typical piRNAs. These data strengthen the idea that *torimochi* is a cluster that produces canonical piRNAs.

As originally shown in Fig. 4, *torimochi* is the most actively translocated transposon in BmN4 cells with extremely high transcription and piRNA production levels and open chromatin structure, thereby representing those transposons that have gained the piRNA production activity in BmN4 cells. To further investigate if *torimochi* has any special features even among those piRNA-producing transposons in BmN4 cells, we have now performed a new analysis. It is known that well-established piRNA clusters in *Drosophila* (e.g., the *42AB* cluster) have a specialized system for transcriptional activation. However, those specialized transcriptional activators such as Rhino (HP1 variant) and Moonshiner (TFIIA variant) are conserved only within the *Drosophila* genus, and thus the transcriptional activation systems of piRNA clusters are likely to be different in different organisms. Keeping this in mind, we asked if the transcription mechanism of *torimochi* is any different from other piRNA-producing transposons in BmN4 cells. Since specific transcriptional activators of piRNAs clusters remain unknown in silkworms (as in many other animals except for *Drosophila*), we decided to differentiate BmN4 cells into adipocytes so that they lose their “germline-ness” (Akiduki et al., 2007). As expected, the expression of the adipocyte marker *BmFABP1* (Fatty Acid-Binding Protein 1) was markedly increased (Fig. 5a), while the expression levels of piRNA-related factors such as *Vasa* were decreased (Fig. 5b). Importantly, transcription of *torimochi* was drastically reduced by adipocyte differentiation (Fig. 5c), whereas most other transposons, including those piRNA-producing transposons in BmN4 cells, remained unrepressed or rather increased by differentiation (Fig. 5c and 5d). These findings suggest that, even among those piRNA-producing transposons in BmN4 cells, *torimochi* has started to gain a specialized, germline-specific transcriptional activation system and thus can be used as a good model as a “young and still growing piRNA cluster.” We will include these data and discussion in the revised manuscript.

Figure 5

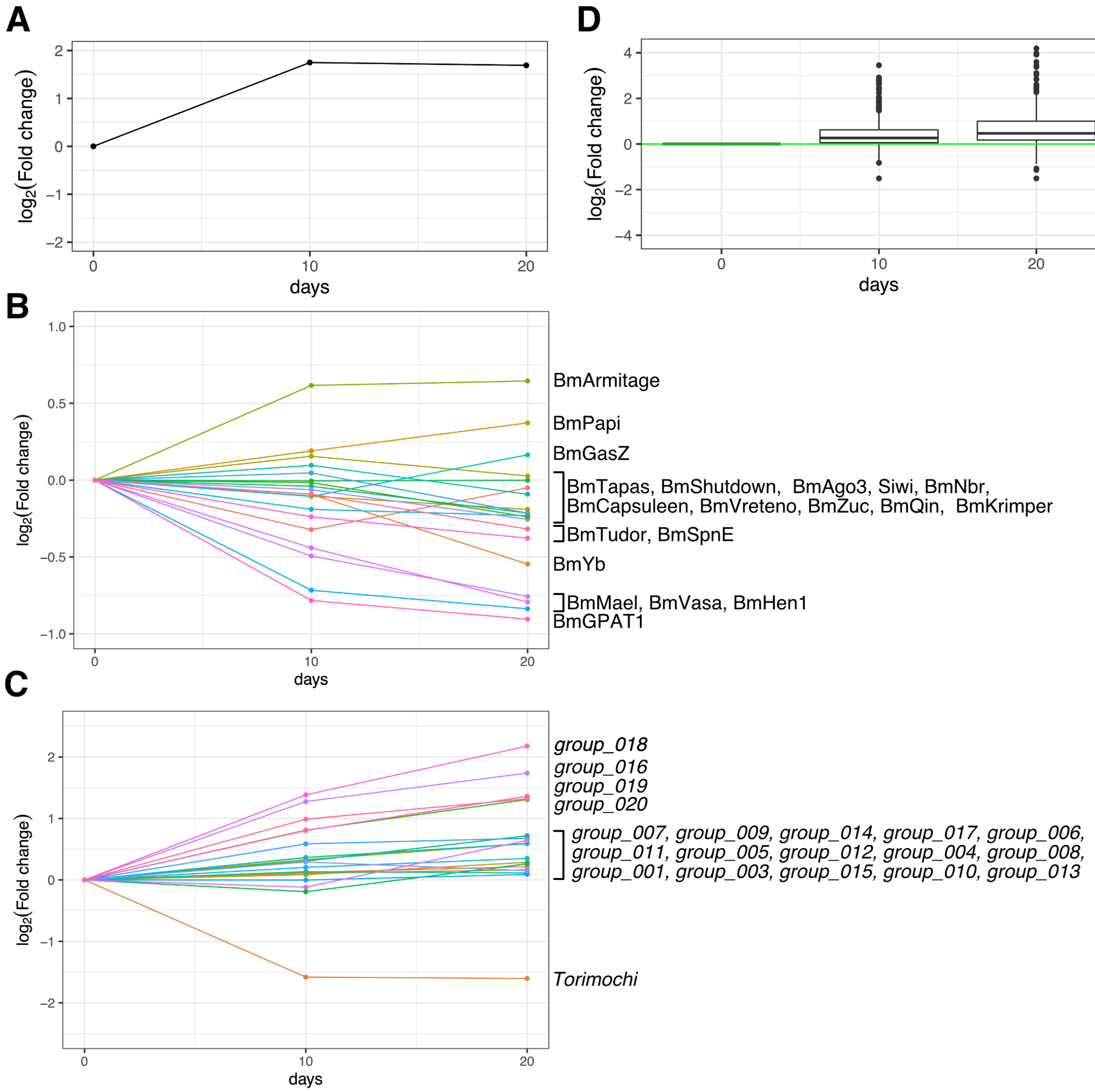

Figure 5 Downregulation of torimochi by differentiation of BmN4 cells into adipocytes.

(A) Relative expression levels of BmFABP1 (Fatty Acid-Binding Protein 1) during differentiation of BmN4 cells into adipocytes, calculated from the RNA-seq data. The expression levels at day 0 (untreated) were used as a baseline. BmFABP1 is upregulated by differentiation.
(B) Relative expression levels of piRNA-related genes during differentiation of BmN4 cells into adipocytes, calculated from the RNA-seq data. The crowded parts are labeled in the order of relative expression levels at day 20. The expression levels at day 0 (untreated) were used as a baseline. Many piRNA-related factors are downregulated by differentiation.
(C) Relative expression levels of the insert groups identified in Figure 4 during differentiation of BmN4 cells into adipocytes, calculated from the RNA-seq data. The crowded parts are labeled in the order of relative expression levels at day 20. The expression levels at day 0 (untreated) were used as a baseline. Torimochi is downregulated by differentiation, whereas other transposons are upregulated.
(D) Box plots of the relative expression levels of transposons during adipocyte differentiation, calculated from the RNA-seq data. 579 transposons with RPKM values greater than 10 in any of the libraries on day0, 10, or 20 were plotted. Center line, median; box limits, upper and lower quartiles; whiskers, 1.5 × interquartile range; points, outliers.

In figure 1F, the positive control (P50T) is missing. Based on the description, this one should show a band, but doesn't or at least doesn't do very clearly. The authors need to repeat this assay.

We agree that the P50T band was quite faint, although it was clearly present at the expected molecular size. We will repeat this assay with more PCR cycles so that the band will appear more clearly.

The authors should perform a qPCR (or similar assay) on the different torimochi loci (and across different strains) to assess their individual transcriptional activity. Generally, showing that torimochi is an active transposable element is crucial to support the claim that it is still expanding.

We have now re-analyzed our RNA-seq data to assess the individual transcriptional activity of different *torimochi* loci. We found that, as expected, *torimochi* mRNAs are a mixture of transcripts from various loci, just like *torimochi*-derived piRNAs. We will include these data in the revised manuscript.

I would also recommend the authors to perform ping-pong analysis on all piRNAs mapping to torimochi. The hypothesis that torimochi acts as a piRNA cluster would be supported showing phased biogenesis, and a lack of a ping-pong signature (i.e., 10A). Please provide evidence that the piRNAs mapping to the different torimochi insertions are not produced via Post Transcriptional Gene Silencing.

We would like to note that silkworms have no homolog of *Drosophila* Piwi, the PIWI protein that is specialized for the phased piRNA biogenesis pathway. Instead, silkworm Siwi participates in both the ping-pong pathway and the phased piRNA pathway (Izumi and Shoji et al., 2020). As expected, *torimochi*-derived piRNAs show both the ping-pong signature and the head-to-tail phasing signature in Trimmer knockout BmN4 cells. We would also like to note that, even in *Drosophila*, dual-strand piRNA clusters (e.g., 42AB) are known to show the ping-pong signature, while uni-strand piRNA clusters (e.g., flamenco) lack it (refs).

Line 265; "Torimochi has the open chromatin structure and can trap foreign transgenes as well as endogenous transposons" - The evidence for "trapping" transposable elements is circumstantial. Transposons are known to insert into each other. One occasion of a transgene inserting in torimochi is not strong enough evidence to support the made claim.

We appreciate the Reviewer's concern. We would like to note that, in the previous paper (Kawaoka et al., 2009), the GFP transgene was inserted into *torimochi* (not once but) at least three times independently; there were three out of eight independent lines that contained the GFP transgene inserted into *torimochi* for piRNA-mediated silencing. This observation highlights the especially efficient "trapping" ability of *torimochi*. We will revise the text to clarify this point.

Please provide a size distribution of all the piRNAs that are mapping on torimochi. In the methods section it is stated that small-RNAs of length 20-42 nt are mapped. This range is too generous as it also includes siRNA on the low end, and other ncRNAs on the long end. Please use the appropriate piRNA size range, i.e., 23-30 nt.

We will be happy to include the size distribution data of all small RNAs mapped to *torimochi*, which shows that only 6% of them are siRNAs (~21 nt) and the majority (82%) of them can be considered as piRNAs (23–32 nt).

Please include the sequences of the newly identified transposon families.

We will be happy to determine the exact sequences of the newly identified transposons in BmN4 cells by PCR and Sanger sequencing and deposit them in a public database.

Minor comments

Line 71-72; "However, it was recently shown that these large piRNA clusters are evolutionarily labile and mostly dispensable for transposon suppression", this is misleading in the context of flamenco since flamenco is essential for transposon suppression. Please rephrase.

We agree that *flamenco* is essential for transposon suppression in the somatic follicle cells in *Drosophila*, and we will rephase the sentence accordingly.

Line 100; "Therefore, torimochi may serve as a model for young piRNA clusters, which are still "alive" and active in transposition, can trap other transposons, and produce de novo piRNAs.". It is unclear how this is evidenced? Would not any transposon be able to "trap" external sequences (e.g., PMID: 33347429). It is unclear to me how torimochi is different from any active transposon that is silenced by the piRNA pathway.

As discussed above, our new data show that *torimochi* is not only a representative of transposons that have gained piRNA-producing activity in BmN4 cells but also a unique transposon that has started to gain a specialized transcriptional activation system as seen in well-established piRNA clusters in *Drosophila*. Therefore, we believe that *torimochi* will serve as a good model for young piRNA clusters.

Line 117; "Therefore, torimochi is not a unique sequence in the genome but should be now interpreted as a gypsy-type transposon" - Even if there is one copy in the genome, torimochi could still is a transposable element.

We agree that, even if there is one copy in the genome, it could still be a transposable element. We will change this part into "Therefore, *torimochi* is not a unique sequence in the genome as was thought in the past but should be now interpreted as a gypsy-type transposon with multiple copies in the genome".

Line 133; "a presumed ancestor of Bombyx mori" - both species are extant, so none of them can be an ancestor of the other.

Yes, *Bombyx mandarina* is also an extant species. We will change the wording to "a wild progenitor of *Bombyx mori*."

Line 135 Change "species" into "strains"?

Yes, "strains" is appropriate and we will change it accordingly

Please provide the coverage for every SNP in figures 2D and 2E. Having an idea of the coverage (i.e., how many reads support this SNP) would strengthen the conclusions made.

We will add a Figure that shows the coverage of each SNPs at the top of the current Figure or as a Supplemental Figure.

Supplementary figure 2I/J; The insert depiction of the GFP cassette is incorrect, it currently is displayed as a small vertical strip, whereas it should be a large block.

We originally intended to show the situation around the GFP cassette for the sake of consistency with Supplemental Figure S2A–H. We will redraw this figure with including the GFP cassette.

Methods: More details are needed on the computational analysis. Please include parameters used for different tools as well as custom scripts. Where multi-mappers used to quantify piRNAs across the torimochi insertions?

We will include precise parameters used for different tools and upload our custom scripts on GitHub.

Display of Supplementary Table 2 and Supplementary Table 3 partially obscured.

We are sorry for the problems caused by the conversion. We will amend them.

Introduction/discussion: I would suggest that the authors also discuss how torimochi could be mis-identified as a piRNA cluster previously.

We will include the following statement to explain why *torimochi* was originally thought as a unique piRNA cluster in the genome. "The silkworm genome published in 2008 had many unassembled regions, which had masked two out of the three *torimochi* copies that we now found to exist in the p50T genome. In other words, the 2008 silkworm genome appeared as if there was only one region to which *torimochi*-derived piRNAs were mappable. Back then, the apparent difference in the chromosomal position of

torimochi between BmN4 cells and silkworm ovaries was thought to be due to a large rearrangement of the corresponding genomic region."

CROSS-CONSULTATION COMMENTS
Reviewer #2 raised three interesting points and the manuscript would be strengthened by addressing these.

We will also fully address the three points raised by Reviewer #2.

Reviewer #1 (Significance (Required)):

Significance

I find the topic both important and timely with the ongoing re-examination of whether piRNA clusters or dispersed euchromatic transposon insertions fuel the piRNA pathway. However, I feel that the current study on torimochi is relatively shallow and descriptive and does not take us much closer to resolving the issue. Re-examining the torimochi cluster is on its own of minor significance, since there are only five publications on torimochi since 2012. However, the current study has potential and torimochi could act as a model to study how piRNAs are produced.

We are grateful to the Reviewer for recognizing the potential importance of our current study. All the comments by the Reviewer were of great help in significantly improving our manuscript. In particular, new Fig. 5 (related to Major Point #1) is an important addition to support the idea that *torimochi* is a young and still growing piRNA cluster, and we thank the Reviewer again for his/her constructive comments.

Reviewer #2 (Evidence, reproducibility and clarity (Required)):

The author performed a straightforward of long read DNA sequencing data, which indicates that torimochi is not a single locus, but a gypsy-like LTR transposon that has massively expanded in BmN4 cells. The data are clear and convincing, and raise a number of interesting questions:

1. The authors present data on single nucleotide polymorphisms in torimochi insertions (Figure 2), but the element can capture transgenes and produce silencing piRNAs. Does the long read data reveal capture of transposon insertions by any of the torimochi elements? Do any appear to be expanding due to recurrent insertion?

In original Fig. S3A, we demonstrated that an endogenous transposon named *mejiro* is indeed inserted into the *torimochi* element . We plan to perform additional long read sequencing and further analyze the data to see if there are other examples of transposon capture events by any of the *torimochi* elements.

2. The data indicate that torimochi is active and transcribed, but also the source of piRNAs that can silence transgenes. Why isn't torimochi silenced by piRNAs derived from the dispersed insertions?

We believe that *torimochi* is indeed being silenced by piRNAs, but just not 100%. The GFP transgene trapped by *torimochi* was also not 100% silenced and some GFP signals were clearly detectable even in the silenced cell lines (Kawaoka et al., 2011). This must be also the case for any other transposons, although the silencing efficiency (the current result of the tug-of-war between transposons and the host's piRNA system) should vary.

3. Comparisons with the silkworm genome indicates that torimochi has been very active since BmN4 were isolated, and the element appears to active now, based on transcription. However, activation could have occurred when the cell line was established. If transposition is ongoing, BmN4 cells maintained as independent stock should have different insertions. This could be tested by sequence analysis of stocks from different labs. This experiment isn't essential to publication, but could be informative.

We thank the Reviewer for raising this important point. Indeed, there exist BmN4 cells that have been independently maintained, and we have now obtained another stock of BmN4 cells from a different lab. We plan to perform long-read sequencing of genomic DNA using these cells to compare the insertion sites of *torimochi*. The results will allow us to determine whether activation of *torimochi* occurred when the cell line was established or its transposition is ongoing. Either result would be informative and helpful to further improve our manuscript.

Reviewer #2 (Significance (Required)):

piRNAs have a conserved role in transposon silencing. In many systems the most abundant piRNAs are derived from distinct chromosomal loci, termed clusters, that are composed of complex arrays of transposon fragments. Available data indicate that these loci can produce trans-silencing piRNAs, and the flam locus is required for fertility and silencing of Gyspsy transposons in flies. However, several major clusters, in flies and mice, are not required for fertility or transposon silencing, and dispersed mobile elements can produce piRNAs. The nature and function of piRNA source loci thus remains to be established. Shoji et al. address that nature of piRNA source loci through a reevaluation of the torimochi cluster In silkworm BmN4 cells. The authors show that torimochi is actually a gypsy-like LTR transposon that has massively expanded in BmN4 cells, and may represent an emerging piRNA clusters, falling between established clusters that look like "transposon graveyards", and single euchromatic insertions that appear to have epigenetically converted to "mini-clusters". The data raise a number of interesting questions, and should stimulate studies in other systems for similar elements.

Revision Plan

We are grateful to the Reviewer for precisely understanding the significance of our current study.

3. Description of the revisions that have already been incorporated in the transferred manuscript

4. Description of analyses that authors prefer not to carry out
